# Inhibition of biofilm formation on the surface of water storage containers using biosand zeolite silver-impregnated clay granular and silver impregnated porous pot filtration systems

**DOI:** 10.1371/journal.pone.0194715

**Published:** 2018-04-05

**Authors:** Phumudzo Budeli, Resoketswe Charlotte Moropeng, Lizzy Mpenyana-Monyatsi, Maggie Ndombo Benteke Momba

**Affiliations:** Department of Environmental, Water and Earth Sciences, Tshwane University of Technology, Arcadia Campus, Pretoria, South Africa; University of Illinois at Urbana-Champaign, UNITED STATES

## Abstract

Development of biofilms occurring on the inner surface of storage vessels offers a suitable medium for the growth of microorganisms and consequently contributes to the deterioration of treated drinking water quality in homes. The aim of this study was to determine whether the two point-of-use technologies (biosand zeolite silver-impregnated clay granular (BSZ-SICG) filter and silver-impregnated porous pot (SIPP) filter) deployed in a rural community of South Africa could inhibit the formation of biofilm on the surface of plastic-based containers generally used by rural households for the storage of their drinking water. Culture-based methods and molecular techniques were used to detect the indicator bacteria (Total coliforms, faecal coliform, *E*. *coli*) and pathogenic bacteria (*Salmonella spp*., *Shigella spp*. and *Vibrio cholerae*) in intake water and on the surface of storage vessels containing treated water. Scanning electron microscopy was also used to visualize the development of biofilm. Results revealed that the surface water source used by the Makwane community was heavily contaminated and harboured unacceptably high counts of bacteria (heterotrophic plate count: 4.4–4.3 Log_10_ CFU/100mL, total coliforms: 2.2 Log_10_ CFU/100 mL—2.1 Log_10_ CFU/100 mL, faecal coliforms: 1.9 Log_10_ CFU/100 mL—1.8 Log_10_ CFU/100 mL, *E*. *coli*: 1.7 Log_10_ CFU/100 mL—1.6 Log_10_ CFU/100 mL, *Salmonella* spp.: 3 Log_10_ CFU/100 mL -8 CFU/100 mL; *Shigella* spp. and *Vibrio cholerae* had 1.0 Log_10_ CFU/100 mL and 0.8 Log_10_ CFU/100 mL respectively). Biofilm formation was apparent on the surface of the storage containers with untreated water within 24 h. The silver nanoparticles embedded in the clay of the filtration systems provided an effective barrier for the inhibition of biofilm formation on the surface of household water storage containers. Biofilm formation occurred on the surface of storage plastic vessels containing drinking water treated with the SIPP filter between 14 and 21 days, and on those containing drinking water treated with the BSZ-SICG filter between 3 and 14 days. The attachment of target bacteria on the surface of the coupons inoculated in storage containers ranged from (0.07 CFU/cm2–227.8 CFU/cm^2^). To effectively prevent the development of biofilms on the surface of container-stored water, which can lead to the recontamination of treated water, plastic storage containers should be washed within 14 days for water treated with the SIPP filter and within 3 days for water treated with the BSZ-SICG filter.

## Introduction

Appropriate sanitation facilities and piped water systems have been reported to be the key to reducing or even eradicating the burden of waterborne diseases[1–4]. However, the establishment of these types of infrastructure could take decades, especially in impoverished rural communities of African countries[5]. Failure to provide adequate safe drinking water in developing countries through centralised systems has led to a renewed interest in point-of-use treatment methods. Decentralised drinking-water systems have been found to be an important aspect in the process of providing safe drinking water to rural and peri-urban areas of developing and transition countries as centralised systems are often deficient or non-existent in these areas[1–8].

Various types of household water treatment and safe storage (HWTSS) systems have been reported in the literature during the past decade. Studies have demonstrated that storage methods and relatively inexpensive home water treatment systems have contributed tremendously to the substantial improvement in the microbial quality of drinking water and the reduction in the risk of diseases and death, even in the absence of improved sanitation[1–4,7,8]. However, factors such as handling practices and the conditions under which the water is stored in homes as well as the types of storage containers have the potential to reverse the benefit gained from these drinking water treatment methods[9,10].

Furthermore, biofilm formation on the inner surface of storage vessels has been reported to offer a suitable medium for the growth of microorganisms and consequently to contribute to the deterioration of drinking water quality in homes[9,10]. Studies have shown high counts of heterotrophic bacteria[11,12] and faecal coliforms [11,13] in stored drinking water, which by far exceeded the limits set for human consumption. Given the right conditions, a small number of microorganisms in water may also provide a seed, which will allow them to multiply in the storage containers. A study by [14] has shown that microorganisms attached on the surface wall of such containers during storage multiplied at the expense of low concentrations of carbon in water. These authors found a direct relationship between the degree of bacterial re-growth on drinking water storage vessels and the storage materials and the quality of the intake water [(such as temperature, turbidity and concentrations of organic nutrient (dissolved organic carbon–DOC)]. A comparison of the quality of drinking water stored in polyethylene (PE) and galvanized steel (GS) containers by the rural communities of South Africa revealed a higher re-growth of total coliforms on PE than on GS containers as the former contained higher DOC concentrations than the latter [14]. However, plastic-based materials are the most widely used for water storage containers in the developing world as rural communities can afford these products. There is therefore a need to improve the quality of drinking water stored in these plastic-based vessels.

Deterioration of drinking water quality during storage is one of the major difficulties not only experienced in decentralised systems, but also by potable water suppliers in centralised systems. The most alarming situation is the occurrence of pathogenic and opportunistic bacteria such as *Legionella* spp., *Pseudomonas*, *Mycobacterium*, *Campylobacter*, *Klebsiella*, *Aeromonas*, *Helicobacter pylori*, *Salmonella* and *E*. *coli* within biofilms [15]. It is therefore important for suppliers of home drinking water treatment technologies to make sure that the technologies provide safe drinking water after treatment and during storage in homes. Although various systems and devices have been extensively reported to improve the quality of water and the health of the population, scant attention has been given to the potential sustainability of these devices and very little guidance is available in terms of maintenance to prevent the development of biofilms during storage. Taking into consideration the deterioration of the stored water and the health risk associated with pathogenic microorganisms within this type of water, this study investigated the efficiency of the household drinking water treatment technologies developed by the Tshwane University of Technology (TUT) in inhibiting the growth of biofilms on the most widely used water storage container prior to their deployment in South African rural communities. We were also able to provide information regarding their potential sustainability as well as guidance in terms of their maintenance.

## Materials and methods

### Description of household drinking water treatment technology

**Fig 1:**
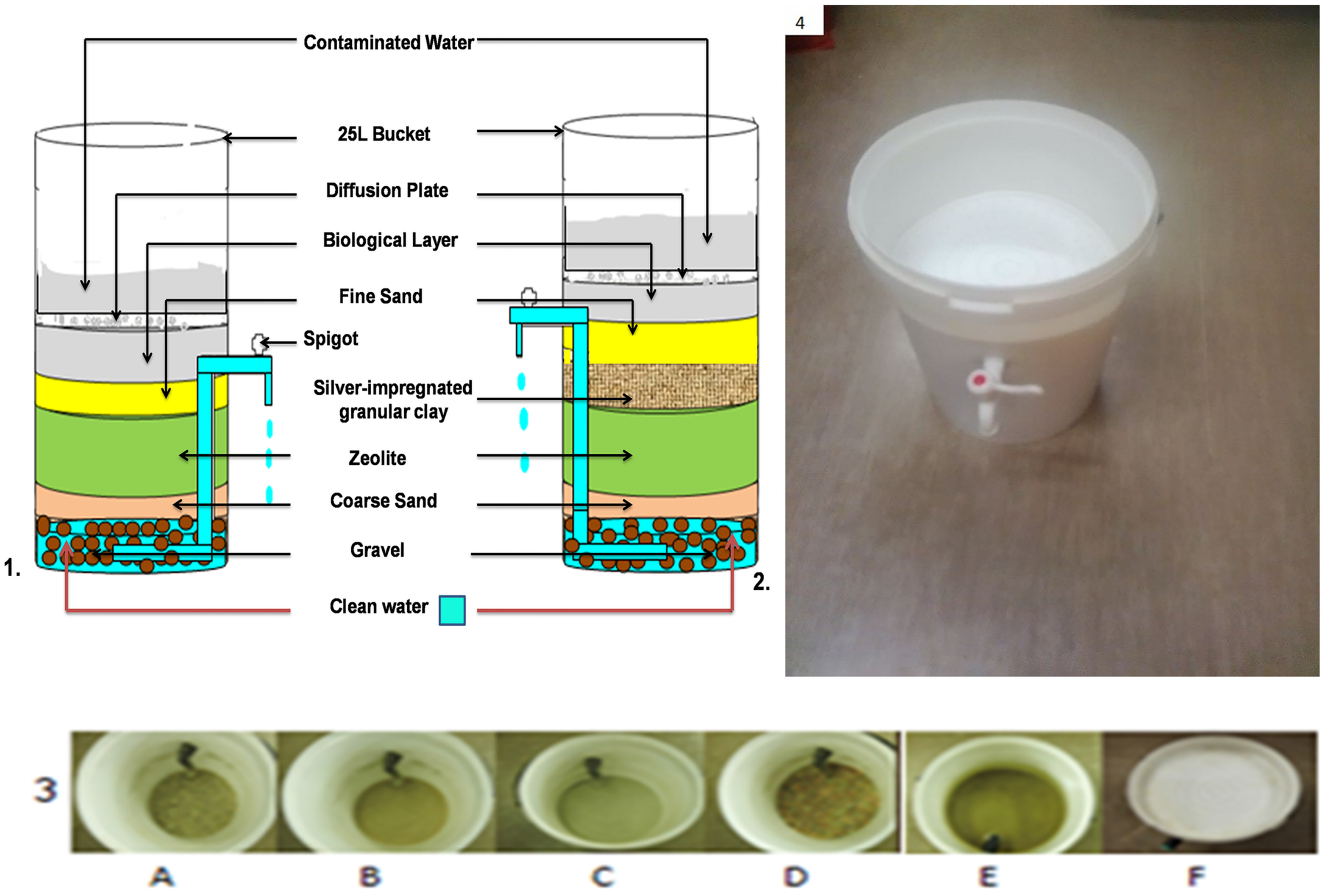
Schematic representation of modified HWTS—(1) BSZ-SICG; (2) modified BSF with zeolite and silver-impregnated granular clay; (3) layers of the modified BSF (A—7 mm gravel; B—0.95 mm coarse sand; C—3 mm natural zeolite; D—silver-impregnated granular clay; E—diffusion plate; and F—0.15 mm fine sand); (4) a complete BSZ-SICG.

**Fig 2:**
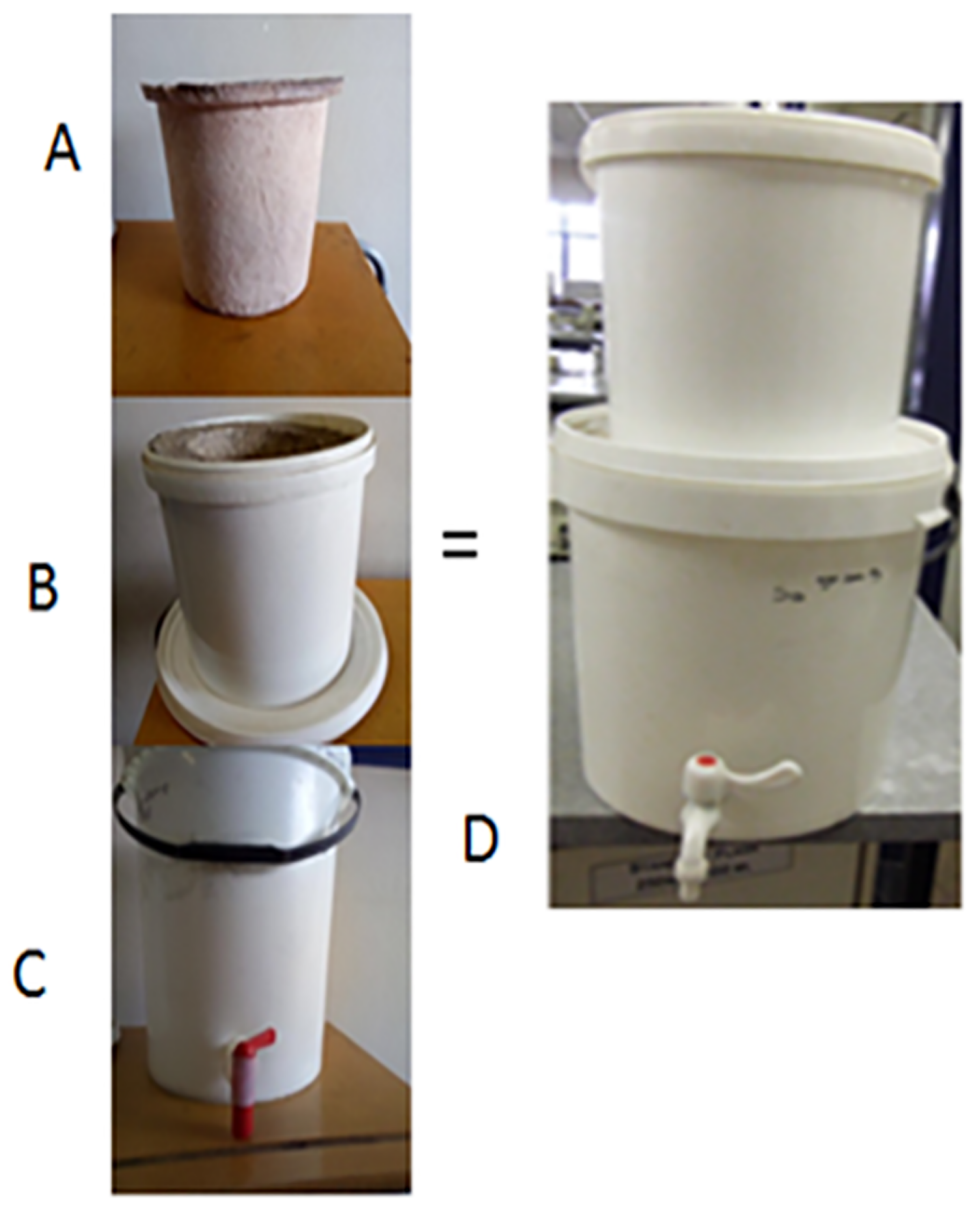
Schematic representation of the SIPP filter—A: silver-impregnated porous pot; B: 10 L bucket with clay pot inside; C: 20 L bucket for the collection of treated water; D: a complete SIPP filter.

**Fig 3:**
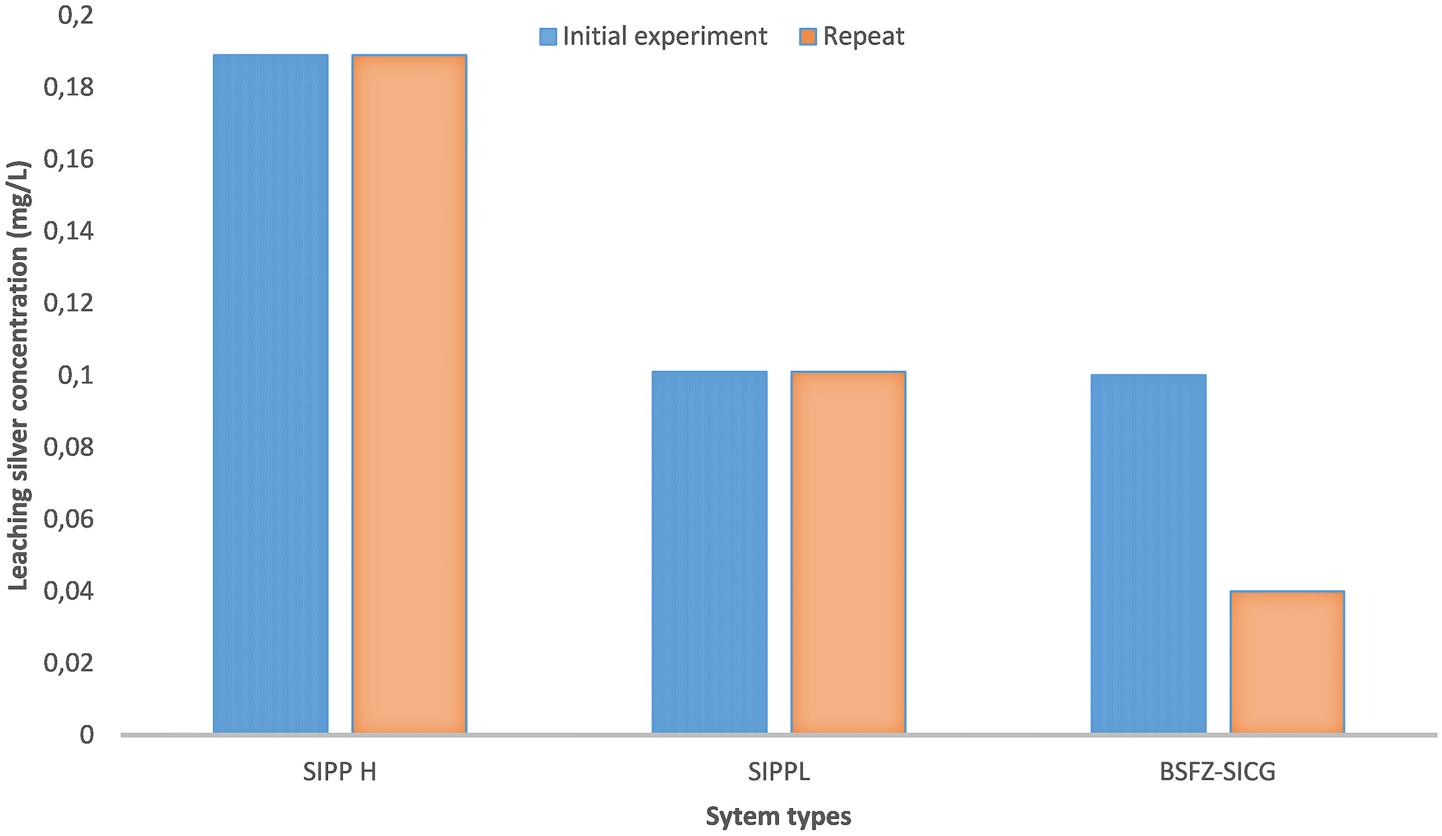
Amount of silver leached from BSZ-SICG, SIPP-H and SIPP-L filters.

Prior to the commencement of the study, ethical clearance was received from Tshwane University of Technology Research and Ethics Committee. In a previous study series by [5,6], it was revealed that a combined biosand and zeolite (BSZ) filter and a silver-impregnated porous pot (SPP) filter manufactured by the TUT Water Research Group could play a major role in providing access to safe drinking water to rural communities. However, prior to being deployed in rural households of the Makwane Village in the Limpopo Province of South Africa, some modifications were required to enhance their performance. The SIPP filter was modified in terms of the flow rate which increased from 2.5 L/h to 27.5 L/h. A layer of silver-impregnated granular clay was added to the BSZ filter to form a BSZ-SICG filter. The flow rate of this filter increased from 19 L/h to 38.6 L/h. With these improved flow rates, both household water treatment systems (HWTS) achieved the required volume of 25 L/person.d. A schematic representation of the cross-section of the initial household BSZ filter is given in Fig 1 and the cross-section of the modified system, called the BSZ-SICG filter, is given in Fig 1. In Fig 2 a schematic representation of the SIPP filter is given. During the study period, two sets of SIPP filters, which had different initial silver concentrations (SIPP-H with 0.18 mg/L and SIPP-L with 0.101 mg/L) were evaluated as shown in Fig 3. The biosand filters comprised the BSZ-SICG filter with an initial silver concentration of 0.1 mg/L in the clay layer and the BSZ filter without the silver-impregnated granular clay layer.

### Experimental study design

The study was conducted in accordance with the Declaration of Helsinki, and approved by the Faculty of Science Research Ethics Committee (FCRE) at the Tshwane University of Technology (TUT), where the study was registered Ref: FCRE 2014/05/20 (SCI) see also S1 File for downloadable consent form. Furthermore, the authorization to conduct the study in Makwane Village was obtained from Elias Motswaledi Municipality’s Manager, the Municipal Councillor and the Committee. The informed consent was obtained from the participants of the selected households prior to commencement of the project. All the project expectations and respective obligations by both the participants and the researcher team were clearly explained to the community using the local language. It is important to note that the participation of the Makwane community in the project was voluntary and the participants were not subjected to risks of any kind because of the project. Due to the lack of cooperation of the householders in terms of the field study requirements to conduct the biofilm study in the homes, this component of the study was discontinued and the biofilm experimental study was then conducted only in a laboratory setting. The experimental study was performed in triplicate.

### Collection and assessment of the intake water quality

Water samples were collected every two weeks between March and June 2015 from a river that serves as the main water source to the Makwane Village, using 500 mL sterile sampling bottles and 50 L sterile water drums. Water samples collected in 500 mL sterile bottles were used as controls to determine the initial concentrations of the target bacteria in the test water, while the 50 L drum water samples were treated using the household devices to ascertain their efficiency in inhibiting biofilm formation on the surface of the storage vessels. Prior to use, the bottles and the drums were washed properly with detergents, rinsed, tightly closed and properly labelled to avoid further contamination. The bottles were autoclaved at 121 °C, while the drums were exposed overnight to the UV light in a laminar flow. The samples were analysed within 24 h after collection for the detection of indicator bacteria (total coliforms, faecal coliforms, *E*. *coli*) and pathogenic bacteria (*Salmonella spp*., *Shigella spp*. and *Vibrio cholerae*) and the determination of physicochemical characteristics of the intake raw water. The pH and turbidity were measured using a pH meter (Metrohm Co. Model 713) and a Hach 2100P portable turbidity meter (Eutech Instruments Turbidimeter TN-100), respectively. The temperature of the water samples was determined using a conductivity meter (Hach Co. Sension7). The physicochemical and microbiological quality parameters of the untreated water were then compared with the standards set by SANS 241 [16]. It is worth noting that most the households in the Makwane Village rely on this water source for drinking and other domestic purposes without any prior treatment.

### Treatment of the intake water and inoculation of plastic-based material slides

Treatment of water samples and the biofilm study were conducted at TUT Water Research Group using both improved BSZ-SICG and SIPP filters and the BSZ filter. Water samples were filtered daily through each device in the laboratory as follows: 10 L/d for the SIPP filter and 20 L/d for the BSZ-SICG and BSZ filters. For each device, the treated water was collected in a 25 L improved storage container with a tap installed 5 cm from the base of this container Fig 1. Plastic-based coupons (like the plastic material of the container commonly used by the community to store their drinking water) with the size of a microscope slide (75 mm x 25 mm x 1 mm) were placed in the lids of sterile Petri dishes, which were suspended in 25 L storage vessels during the study period. The tiny holes in the lids of the Petri dishes allowed the treated water to freely pass through the lids and kept them floating in the storage vessels. During the study period, the coupons were also suspended in a storage vessel which was used as a control and it contained raw water without treatment. Prior to use, all these vessels and coupons were thoroughly washed with sterile distilled water and placed overnight under a UV light with laminar flow. Their sterility was microbiologically tested to prevent any contamination of the treated water.

To mimic the situation in rural areas, the filters were run daily while decreasing the quantity of the treated water in the storage containers through the connected taps in case the containers became full. The raw water in the control vessel was also replaced daily. To establish the performance of the household drinking water treatment technologies in inhibiting the formation of biofilms and to determine the relevant time period for the cleaning of collection vessels, the storage containers were not washed before the apparent formation of biofilms on the surface of coupons. Once this occurred, new coupons were re-suspended in the washed containers and these series of experiments were repeated twice during the study period. This was imperative in making the necessary recommendations in terms of hygienic conditions of these vessels during storage. The storage containers were opened only under the UV laminar flow to aseptically remove two coupons after Day 1, Day 2, Day 3, Day 7, Day 14 and Day 21. The physicochemical characteristics of the stored untreated were also determined concomitantly on the days of the coupon removals.

### Microbiological analysis of the intake and treated water samples and inoculated coupon slides using culture-based methods

The target bacteria were isolated from the raw intake water and treated water samples and on the surface of container stored-water slides according to standard procedures [17] using selective media. For the intake and treated water samples, the colonies were counted and recorded as CFU/100 mL. For each household drinking water device and the control storage vessel, one of the two coupons was immersed in 50 mL sterile centrifuge tubes with 30 mL sterile saline water. The attached bacteria were released from the coupons by vortexing for 2 min using a vortex mixer. The following equation was used to calculate the number of indicator and presumptive pathogenic bacteria found on the coupons and the counts were expressed in CFU/cm^2^:

$$\text{Attached}\;\text{bacterial}\;\text{counts}\left(\text{CFU}/{\text{cm}}^{2}\right)=N\times D/\text{surface}\;\text{of}\;\text{coupon}\;\text{slide}$$

where *N* = average number of colonies and *D* = dilution factor.

#### Heterotrophic plate count bacteria

Heterotrophic plate count agar (Merck, SA) was prepared according to the manufacturer’s instructions. In brief, the spread-plate technique was applied whereby 100 μL of water sample was spread across the surface of a nutrient agar plate and incubated at a temperature of 35°C for 48 h [17]. After incubation, plates were removed from the incubator, and the number of colonies was counted and recorded as CFU/100 mL for the intake raw water. The number of attached bacteria was calculated using the equation given above. The attached HPC bacterial counts were recorded as CFU/cm^2^.

#### Total and faecal coliforms

The water samples were analysed for the presence of total and faecal coliforms bacteria using the membrane filter technique as described in Standard Methods [17]. Briefly, agar plates were prepared according to the instructions of the manufacturers; 100 mL of raw water or 100 mL of treated water samples was filtered through a 0.45 μm pore-size membrane, and the membrane filter paper was then placed on M-Endo agar (Acumedia, USA) and M-FC agar (Acumedia, USA) plates for total and faecal coliforms, respectively. Plates were incubated at 37°C and 44°C for 24 h, respectively. All the samples were tested in duplicate. Colonies were enumerated and recorded as CFU/100 mL for the intake raw water. The counts of attached total or faecal coliform bacteria were calculated using the equation given above and reported as CFU/cm^2^.

#### Pathogenic bacteria

The isolation and detection of pathogenic bacteria (*E*. *coli*, *Salmonella* spp and *Vibrio* spp) were performed using the membrane filter technique as described in Standard Methods [17]. MacConkey agar (Merck, SA), XLD agar (Merck, SA) and TCBS agar (Merck, SA) were used as a culture medium and the plates were incubated at 37°C overnight. Colonies were enumerated and recorded as CFU/100 mL for the intake raw water. The counts of attached pathogenic bacteria were calculated using the equation mentioned above and they were reported as CFU/cm^2^.

### Molecular identification of presumptive pathogenic bacteria isolates

Following the isolation and detection of target pathogenic bacteria, no presumptive pathogenic bacteria were detected in treated water and on the surface of coupons. Consequently, five (5) characteristic colonies for presumptive *Salmonella* spp., *Shigella* spp. and *Vibrio* spp. were randomly selected only from different plates for the intake water source and transferred onto the selective media by the streak-plate method and incubated at 36°C ± 1°C for 24 h. Furthermore, the colonies were purified by the same methods at least three (3) times using nutrient agar (BioLab Inc.) and thereafter they were sub-cultured onto their respective selective media three times prior to being used for molecular identification.

### Extraction of the total genomic DNA

Individual isolates were harvested and grown in a nutrient broth, followed by incubation at 37°C for 24 h. Thereafter the pellets were washed with nuclease-free water. The total genomic bacterial DNA was extracted using boiling method as described by [18]. The quality and quantity of the isolated nucleic acids were determined using the NanoDrop 2000^™^ spectrophotometer (Thermo Scientific) and agarose electrophoresis (BioRad).

### Amplification of presumptive pathogenic *E*. *coli* isolates

PCR was performed with a 25 μL reaction mixture containing 2.5 μL of template DNA, 12.5 μL of master mix DreamTaq^™^ DNA polymerase (2 X DreamTaq^™^ Green Buffer, dATP, dCTP, dGTP, and dTTP, 0.4 mM each, and 4 mM MgCl_2_) and 0.2 μL of each primer (Table 1). Nuclease-free water was added to obtain a final volume of 25 μL. The amplification cycles consisted of an initial DNA denaturation at 94°C for 7 min, followed by 39 cycles of denaturation at 94°C for 30 seconds, primer annealing at 57°C, for 30 seconds, extension at 72°C for 1 min, and a final extension at 72°C for 10 min. Negative controls consisting of the DNA template with nuclease-free water (Inqaba, SA), were included in all PCR runs. DNA extracted from *E*. *coli* ATCC 25922 (Quantum Biotechnologies, SA) was used as a positive control. PCR products (8 μL) were evaluated with a 1.5% (wt/vol) agarose gel (Life Technologies, SA) at 120 mV for 60 min. A molecular marker (100 bp DNA ladder; Inqaba, SA) was run concurrently. All results were captured using a gel documentation system (Syngene, Cambridge, U.K.).

**Table 1:** Oligonucleotides used in this study for amplification of pathogenic genes of *E*. *coli* strains [30].

Primer name	Sequences 5’to 3’	Target genes	Size
**EHEC-423**	F-TGGAAAAACTCAGTGCCTCT-	Stx1	423bp
	R- CCAGTCCGTAAATTCATTCT-		
**EPEC-300**	R- GGAATCAGACGCAGACTGGTAGT-	bfpA	300bp
	F- GGAAGTCAAATTCATGGGGGTAT-		
**ETEC-187**	F- GCTAAACCAGTAGAGGTCTTCAAAA-	ST	187bp
	R- CCCGGTACAGAGCAGGATTACAACA-		
**EIEC-508**	R- CACACGGAGCTCCTCAGTC-	IpaH	508bp
	F- CCCCCAGCCTAGCTTAGTTT-		
**lEAEC-215**	R- ACGACACCCCTGATAAACAA-	aaic	215bp
	F- ATTGTCCTCAGGCATTTCAC-		

### Amplification of presumptive *Salmonella* and *Shigella* isolates

PCR amplification was performed in a final volume of 25 μL consisting of 12.5 μL DreamTaq^™^ master mix (Inqaba Biotech, Pretoria, SA), 0.5 μL solution of each primer and 2.5 μl of the DNA template. Nuclease-free water was added to a final volume of 25 μL and subjected to 30 cycles for amplification in a thermocycler (LASEC, Pretoria, SA). All the primer sequences, size of PCR amplicons and PCR conditions that were used in this study are presented in Table 2. The cycling conditions were as follows: heat denaturation 94°C for 4 min, followed by 30 cycles at 94°C for 1 min, annealing at a temperature of 55°C for 1 second, extension at 72°C for 1 min and final extension at 72°C for 10 min. DNA extracted from *Salmonella* ATCC 14028 and *Shigella dysenteriae* 11835 (Quantum Biotechnologies, SA) was used as positive controls. The PCR products were analysed by electrophoresis on 2.0% w/v agarose gel (Merck, SA) at 100 V for 60 min. A molecular marker (100 bp DNA ladder; Inqaba, SA) was run concurrently. All results were captured using a gel documentation system (Syngene, Cambridge, U.K.).

**Table 2:** Oligonucleotides used in this study for amplification of pathogenic genes of *Salmonella* and *Shigella* spp [31].

Organism	Target gene	Primers	Sequence 5’ to 3’	Amplicon size
***Shigella* genus**	ipaH1	ipaH1	F GTTACCTGTACTCCCTGCTT	204 bp
			R CTCAACTTCAGCAGATACCA	
***Shigella boydii***	Wzy	Wzy	F ACCAAGGAGTTGTTCATGA	305 bp
			R GAAGCCCTGGTAAAGTGC	
***Shigella sonnei***	wbgZ	wbgZ	F ATGTTGCTAATACCAGTTGG	460 bp
			R TAGAGAGAACTTCACACGGT	
***Shigella flexneri***	ipaH	ipaH	F TGAGAATTTGCCTCCACA	595 bp
			R CTAGCCTTCCTTGTGCAA	
***Salmonella enterica***	invA	invA	F GAGGAAAAAGAAGGGTCG	780 bp
			R CTAGCCTTCCTTGTGCAA	

### Amplification of presumptive *V*. *cholerae* isolates

About 2 μL of bacterial DNA was used for multiplex PCR amplification. The PCR reaction mixture contained 10 μL of DreamTaq^™^ master mix (Inqaba Biotech, Pretoria, SA), 0.75 μL of each primer, and nuclease-free water was added to make up the final volume of 20 μL. All primers used in this study are listed in Table 3. The cycling conditions were as follows: heat denaturation step at 94°C for 3 min; 35 cycles of denaturation at 94°C for 1 min, annealing at 55°C for 1.5 min and extension at 72°C for 2.5 min followed by final extension at 72°C for 10 min. DNA extracted from *V*. *cholerae* ATCC 10259 (Quantum Biotechnologies, SA) was used as positive control. The PCR products were analysed by electrophoresis on 2.0% w/v agarose gel (Merck, SA) at 100V for 60 min. A molecular marker (100 bp DNA ladder; Inqaba, SA) was run concurrently. All results were captured using a gel documentation system (Syngene, Cambridge, U.K.).

**Table 3:** Oligonucleotides used in this study for amplification of genes of *Vibrio* spp [32].

Organism	Target gene	Primers	Sequence 5’ to 3’	Amplicon size
***V*. *parahaemolyticus***	Flae aceace	VPAR	F CACTCACCTCTCTCGAACTC	400 bp
			R TAGCGTTGTTCGGAATTACT	
***V*. *fluvialis***	ompW	VFLU	F TATACTATCCGACGACTGGC	250 bp
			R TTCGTAGATATTCGGAGGAA	
***V*. *vulnificus***	SodB	VVUL	F ATTTCACCTCTACACTCGGA	350 bp
			R TAGCGTTGTTCGGAATTACT	
***V*. *cholerae***	rfb	VCHO	F TATACTATCCCGGAGGAACAC	600 bp
	ctxA		R ATATTTCACCTCTCTCGAACTC	
***V*. *proteolyticus***	toxR	VPRO	F CCTCTCTCGAACTCCGGAG	150 bp
			R GTTGTGGAATTACTCGGAGGAA	

### Visualisation of biofilm formation on the surface of inoculated coupon slides

The remaining coupons removed from the treated water and the control storage vessels were rinsed with sterile Milli-Q water for 30 s to remove any unattached cells. Thereafter the coupons were fixed and treated sequentially with 20 mL of 2.5% glutaraldehyde (30 min); 0.15 M phosphate-buffer (3 min x 15 min); 50% ethanol (1 min x 15 min); 70% ethanol (1 min x 15 min); 90% ethanol (1 min x 15 min) and 100% ethanol (3 min x 15 min). They were dried in a critical point dryer for 3 h. Finally, the dried samples were coated with gold plasma so as to make them visible under the scanning electron microscope (SEM) (JEOL JSM 840).

### Monitoring of silver leach levels

The leaching of silver in water filtered by the SIPP and the BSZ-SICG filters was monitored monthly for the duration of the study. The Spectro Arcos ICP spectrometer (Spectro, RSA) was used to detect and determine the concentration of silver in treated water samples.

### Statistical analysis

Biofilm formation data obtained on the interior surfaces of water storage containers containing raw water and water filtered through the various household drinking water systems were analysed utilising the statistical software. Comparisons for means were carried out using a one-way analysis of variance (ANOVA), while tests for correlation between attached bacterial counts and turbidity values were carried out using the Pearson’s correlation coefficient index.

## Results

### Physical and chemical characteristics of intake water, stored-untreated and -treated water samples

The physicochemical characteristics of the raw water before and after treatment were compared with the South African National Standard (SANS) [16]. The intake raw water samples prior to storage had a mean pH ranging between 7.6 and 8.2; these values were within the range (6.5–8.5) set by SANS 241 guidelines. The highest pH of 8, 2 was noted in June 2015, followed by May (pH 7.9). A similar pH of 7.6 was recorded in March and April 2015. The mean temperatures of the intake raw water samples ranged between 19.6 and 24.8°C, which were also within the limits set for drinking water. While March (24.8°C) and April (23.1°C) showed higher temperature values, lower temperatures were observed in May (19°C) and in June (19.6°C).

The mean turbidity values of the raw intake water ranged between 75 and 160 NTU, which by far exceeded the guideline limits set by the South African National Standard SANS 241 for settled water the mean turbidity was found to be 5 NTU and for filtered water prior to disinfection it was <1 NTU. Overall, the highest turbidity value (with an average of 160 NTU) was found in April 2015, followed by March (156 NTU) and May (122 NTU). The lowest turbidity value was observed in June.

Table 4, illustrates the physicochemical characteristics of untreated and treated water samples during the storage period. After the inoculation of the coupons, there was no major increase in the pH of the stored raw water and treated water. In general, the pH values ranged between 7.0 and 8.1, which were also within the limits set by the WHO and SANS 241. Although the mean temperature values for both untreated water and treated water were also within the recommended limits, there were variations according to the types of stored water. Untreated stored water had the highest temperature values ranging between 24.8 and 25.1°C, compared to the treated water. For the water treated by the SIPP filter, the temperature ranged between 21.8 and 23°C, although slight differences were noted between the water treated by the SIPP-H filter and the water treated by the SIPP-L filter, which showed a slight gradual increase in temperature from day 1 (21.8°C) up to day 21 (22.9°C). The temperature of the water treated by the BSZ-SICG filter fluctuated between 19.8°C and 21°C. In spite of these fluctuations, the water treated by this filter had the lowest temperature compared to the water treated by other filters. For the BSZ filter, the water temperature ranged between 19.9 and 25.1 0C, although fluctuations occurred from time to time. There was a gradual increase in the turbidity values of the untreated water from day 1 (mean turbidity value: 158 NTU) up to day 21 (mean turbidity value: 188 NTU) and these values by far exceeded the limit set by WHO guidelines (5 NTU). In general, all the household filtration technologies resulted in significant reduction of the turbidity of the water although there was a slight progressive increase in turbidity values from day 1 up to day 21. Compared to the other filters, the SIPP filters produced drinking water with the lowest turbidity values which were within the recommended limits (SANS 214: < 1 NTU; WHO: 5 NTU), especially between day 1 and day 7. Within the two SIPP filters, lower turbidity values were recorded in drinking water produced by the SIPP-H filter (mean turbidity range: 0.2–2.6 NTU) compared to the SIPP-L filter (mean turbidity range: 0.35–3.0 NTU). Drinking water produced by the BSZF-SICG filter was found to have acceptable turbidity values ranging between 0.66 and 5.1 NTU during the study period; however, a lower turbidity limit of < 1 NTU as set by SANS 241 was recorded between day 1 and day 3. With the exception of day 1, the water produced by the BSZ filter was found to have turbidity values (range of mean turbidity values: 2.8–10.6 NTU) above the recommended limits set by SANS 241.

**Table 4:** Average physicochemical analysis values of untreated and treated water samples (D = days).

Water sources	pH						Temperature (°C)						Turbidity (NTU)					
Time (Days)	D1	D2	D3	D7	D14	D21	D1	D2	D3	D7	D14	D21	D1	D2	D3	D7	D14	D21
Raw	7.4	7.7	7.6	7.8	8.1	8.2	24.9	24.8	25.0	25.1	24.9	25.1	158	160.5	160	166	171	188
SIPP-H	7.4	7.8	8.1	8.0	7.9	7.5	22.0	22.1	22.3	23.1	22.5	23.0	0.2	0.3	0.38	0.65	1.8	2.6
SIPP-L	7.8	7.9	7.9	8.0	7.8	7.0	21.8	22.0	22.1	22.8	22.3	22.9	0.35	0.33	0.45	0.78	2.1	3.0
BSZ-SICG	7.9	8.0	7.9	8.0	8.0	7.8	19.8	19.9	20.4	19.8	21.0	20.8	0.66	0.85	0.95	1.6	3.4	5.1
BSF-Z	7.0	8.0	7.9	7.8	8.1	8.0	19.9	20.5	24.8	25.1	24.9	25.1	2.8	5.6	5.8	6.1	8.0	10.6

### Leaching of silver ions from SIPP and BSZ-SICG filters during storage

During the study period, the leaching of silver ions from both SIPP-H (0.189 mg/L) and SIPP-L (0.100 mg/L) filters into the treated water remained constant in both initial and repeat experiments. However, for the BSZ-SICG filter, a gradual decrease in silver concentration was noted in treated water during the repeat experiment. The silver ion concentration decreased from 0.101 mg/L to 0.045 mg/L.

### Microbial quality of intake water samples and inoculated coupon slides

**Fig 4:**
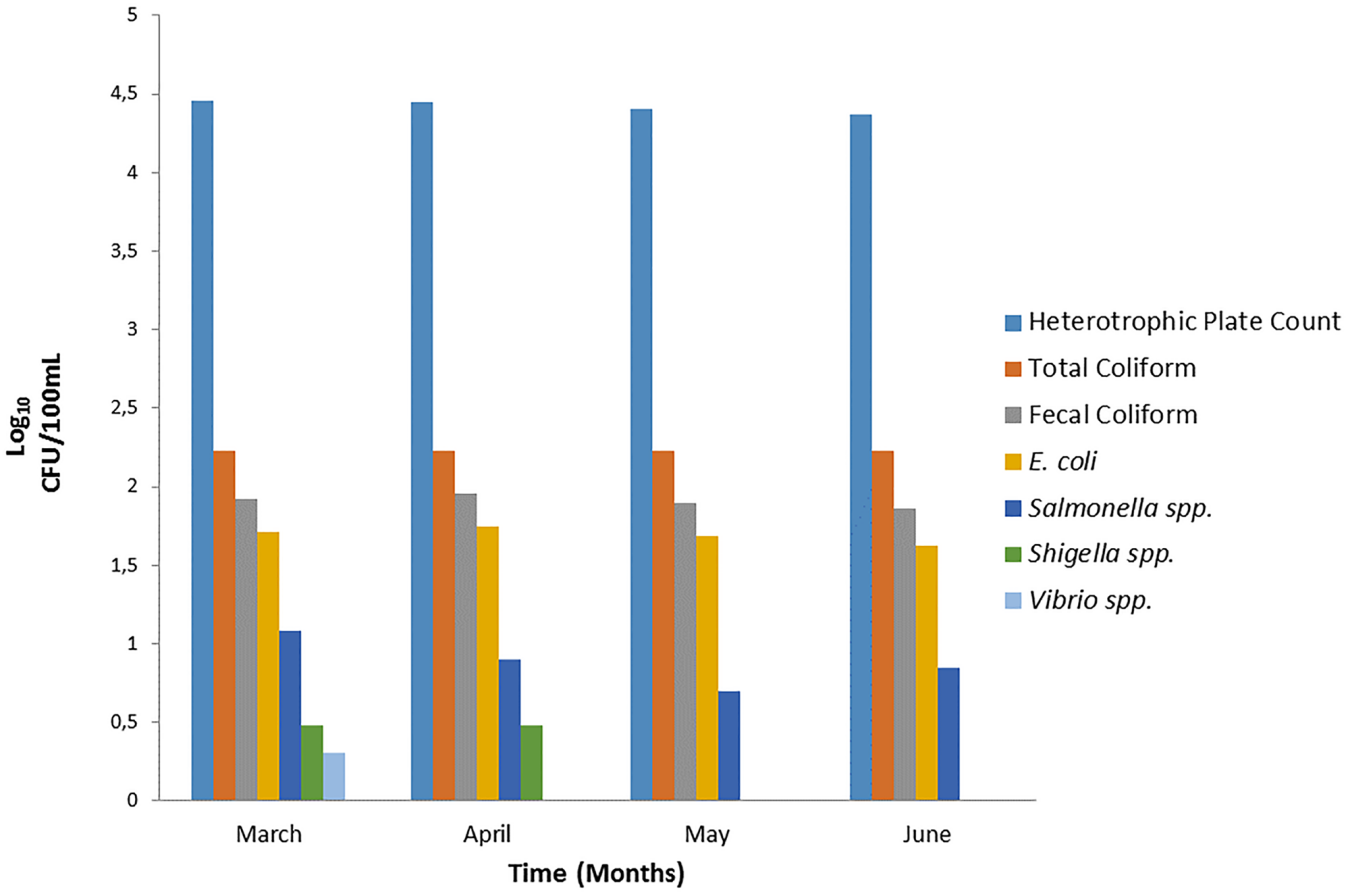
Monthly observations on prevalence of indicator and presumptive pathogenic bacteria in surface water that serves as a drinking water source in Makwane community.

Fig 4, depicts the bacterial quality of drinking water of the Makwane Village using culture-based methods. Results revealed high counts of heterotrophic plate count (HPC) bacteria ranging between 4.4 and 4.3 Log_10_ CFU/100 mL from March to June 2015. The total coliform (TC) counts ranged between 2.225 Log_10_ CFU/100 mL and 2.114 Log_10_ CFU/100 mL, while faecal coliform counts ranged between 1.9 Log_10_ CFU/100 mL and 1.8 Log_10_ CFU/100 mL. Out of all the pathogenic bacteria enumerated, *E*. *coli* was found to be more prevalent with the counts ranging from 1.7 Log_10_ CFU/100 mL to 1.6 Log_10_ CFU/100 mL, followed by *Salmonella* spp., which had counts ranging from 1.079 Log_10_ CFU/100 mL to 0.8 Log_10_ CFU/100 mL. *Shigella* spp. and *Vibrio* spp. were the least detected with their counts ranging from 0 Log_10_ CFU/100 mL to 0.4 Log_10_ CFU/100 mL. With the exception of faecal coliforms, the highest bacterial counts were generally found in March and the lowest in June.

**Fig 5:**
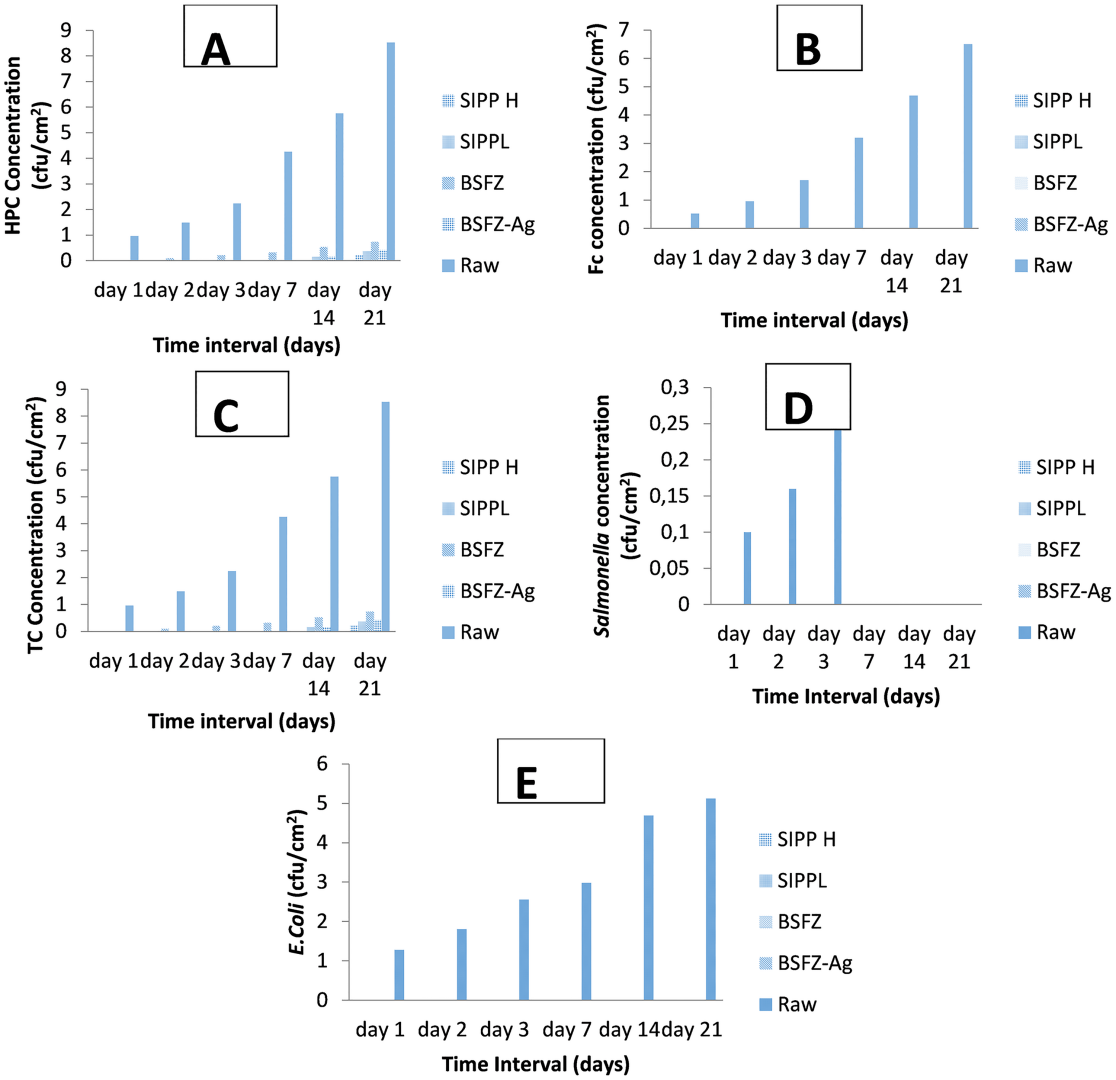
Indicator bacteria and pathogenic presumptive bacteria attached on the surfaces of coupons inoculated in untreated water and treated water produced by various household drinking water treatment systems during the storage period—A: heterotrophic plate count; B: faecal coliforms; C: total coliforms; D: *Salmonella*; and E: *E*. *coli*.

Fig 5, illustrates the attachment of target bacteria on the surface of the coupons inoculated in storage containers. During the storage period, a gradual increase in the average counts of HPC (2.3–5.76 CFU/cm^2^), total coliform bacteria (0.48–4.26 CFU/cm^2^) and faecal coliforms (0.26–3.2 CFU/cm^2^) was apparent on coupons inoculated in untreated water from the first day to the last day of the storage period (Fig 5A, 5B and 5C). A similar trend was noted on coupons inoculated in the treated water produced by BSZ filter (HPC: 20.2–227.8 CFU/cm^2^) and total coliforms (5–35.4 CFU/cm^2^) with the exception of the first day of storage where no evidence of attached bacteria on coupons was observed (Fig 5A and 5C). Attachment of indicator bacteria occurred on coupons inoculated in treated water produced by SIPP-L (HPC: 0.29–0.37 CFU/cm^2^, and total coliform: 0.08–0.1 CFU/cm^2^) and BSZ-SICG (HPC: 0.26–0.48 CFU/cm^2^ and total coliform: 0.07–0.21 CFU/cm^2^) filters between day 14 and day 21 (Fig 5A and 5C). As to the coupons inoculated in the treated water produced by SIPP-H, the indicator bacteria adhered on coupons only after 21 days of storage (HPC: 0.21 CFU/cm^2^ and total coliform: 0.1 CFU/cm^2^) (Fig 5A and 5C). No faecal coliform bacteria were detected on coupons inoculated in the treated water produced by any of the filters (Fig 5B). Among the target pathogenic bacteria, only presumptive *Salmonella* spp. (0.1–0.16 CFU/cm^2^) (Fig 5) and presumptive *E*. *coli* (0.64–2.56 CFU/cm^2^) were detected on the surface of coupons inoculated in vessels containing untreated water during the storage period, while no evidence of their attachment was shown on coupons inoculated in treated water produced by SIPP-H, SIPP-H, BSZ-SICG and BSZ filters (Fig 5E).

As illustrated in Table 5, results obtained from molecular studies confirmed that the selected colonies of presumptive *Salmonella* and *E*. *coli* detected in surface water samples and on the surface of coupons inoculated in the vessel containing stored untreated water were definitely *Salmonella* Typhimurium and *E*. *coli* (EPEC, ETEC, EHEC and EAEC).

**Table 5:** Enteropathogenic bacteria confirmed by PCR from surface water in Makwane community and storage vessels.

Organisms isolated	Presence/Absence
EHEC	+
EPEC	+
ETEC	+
EAEC	+
*Salmonella* Typhimurium	+
*Salmonella enterica*	-
*Shigella* spp.	-
*Vibrio* spp.	-

Analysis of variance (ANOVA) was performed to compare the efficiency of the five devices in inhibiting the bacterial regrowth and data are presented in Table 6. Statistical analysis revealed no significant differences in biofilm formation on coupons inoculated in SIPPH, SIPPL and BSFZ-SICG as these treatments were found to be similar in inhibiting bacterial regrowth (*p*>0.05) when compared to other devices. However, significant differences in biofilm formation were found between SIPPH and raw water in terms of total coliforms and *Salmonella* attachment on coupons. There were also significant differences in biofilm formation in terms of fecal coliforms on coupons exposed to raw water and other silver embedded systems. The performance of raw is significantly different from all the devices (*p*<0.05).

**Table 6:** ANOVA to compare performance of devices across all environmental water source.

Water sources	(*p*)	HPC	FC	TC	*E*. *coli*		*Salmonella*
SIPPH vs SIPPL	*P*	0.0000	NG	1.0000	NG		NG
SIPPH vs BSFZ	*P*	0.9763	NG	1.0000	NG		NG
SIPPH vs BSFZ-SICG	*P*	0.9914	NG	0.9999	NG		NG
SIPPH vs RAW	*P*	0.1458	0.0000	0.0000	0.9914		0.0513
SIPPL vs BSFZ	*P*	0.9762	NG	0.0000	NG		NG
SIPPL vs BSFZ-SICG	*P*	0.9913	NG	0.9996	NG		NG
SIPPL vs RAW	*P*	0.1456	0.0000	1.0000	0.9914		0.0513
BSFZ vs BSFZ-SICG	*P*	0.9999	NG	0.9996	NG		NG
BSFZ vs RAW	*P*	0.3817	0.0000	1.0000	0.9914		0.0513
BSFZ-SICG vs RAW	*P*	0.3116	0.0000	0.9999	0.9914	0.0513	

All the target bacteria attached on coupons inoculated in the untreated water exhibited strong positive correlations, except for *Salmonella* that showed a negative correlation (*r* = -0.6906). The *p*-values showed significant differences between all other attached bacteria and the length of storage of the untreated water, excepted for *Salmonella* (*p* = 4.98412). For the treated water sources, correlations were found only between HPC and total coliforms attached on coupons and the length of the storage. Apart from HPC attached on coupons immersed in water treated by the BSF-Z filter, the *p*-values showed no significant differences for other treated water (Table 6). As no growth of other target bacteria was observed in treated water as well as on the surface of the coupons, the significant difference and the correlations could not be established.

As seen in Table 7, positive strong correlations were noted between the turbidity and all the target bacteria attached on coupons inoculated in untreated water, except for *Salmonella* that showed a negative correlation (*r* = -0.6428). Statistical evidence also showed significant differences between the turbidity of the untreated water and HPC, total coliforms and *E*. *coli* attached on coupons.

**Table 7:** Pearson’s correlation values showing relationship between the attached microorganisms from the coupons inoculated in different sources (untreated water, water treated with SIPP, water treated with BSZ and water treated with BSZ-SICG) and Turbidity (*r*—Correlation; *p*<0.05; NG: no growth of bacteria on coupons).

Water sources	Correlation coefficient based on turbidity	HPC	Total coliform	Faecal coliform	*E*. *coli*	*Salmonella*
Untreated water	r	0.9716	0.9693	0.9554	0.8954	-0.6428
	p	0.0059	0.0100	0.0035	0.0004	8.89E-10
SIPP-H	r	0.8028	0.8028	NG	NG	NG
	p	0.0197	5.3200	NG	NG	NG
SIPP-L	r	0.9831	0.9713	NG	NG	NG
	p	0.9831	0.9713	NG	NG	NG
BSF-Z	r	0.9002	0.9702	NG	NG	NG
	p	0.9002	0.9702	NG	NG	NG
BSZ-SICG	r	0.8911	0.8824	NG	NG	NG
	p	0.8911	0.8824	NG	NG	NG

NG: no growth of bacteria on coupons

In all treated water sources, strong positive correlations were found between turbidity and HPC and total coliforms attached on coupons, while no growth of faecal coliforms, *E*. *coli* and *Salmonella* were observed on the surface of coupons as well as in the treated water. Consequently, the correlation could not be established for these bacteria. Table 8 depicts the correlations between the microorganisms attached on coupons inoculated in different sources (untreated water, water treated with SIPP, water treated with BSZ and water treated with BSZ-SICG) and the length of storage (Days 1–3, Day 7, Day 14 and Day 21).

**Table 8:** Pearson’s correlation values showing relationship between the attached microorganisms from the coupons inoculated in different sources (untreated water, water treated with SIPP, water treated with BSZ and water treated with BSZ-SICG) and the length of storage (Days 1–3, Day 7, Day 14 and Day 21).

Water sources	Correlation coefficient	HPC	Total coliform	Faecal coliform	*E*. *coli*	*Salmonella*
Untreated water	*r*	0.996122	0.9897	0.9890	0.9643	-0.6906
	*p*	0.0011	0.0454	0.0174	0.0026	4.9841
SIPP-H	*r*	0.8011	0.8011	NG	NG	NG
	*p*	0.0731	1.5840	NG	NG	NG
SIPP-L	*r*	0.9562	0.9543	NG	NG	NG
	*p*	0.6750	2.7824	NG	NG	NG
BSF-Z	r	0.9332	0.9840	NG	NG	NG
	p	0.0039	5.5446	NG	NG	NG
BSZ-SICG	r	0.9656	0.9453	NG	NG	NG
	p	0.9368	4.9714	NG	NG	NG

*r*—Correlation; *p*<0.05; NG: no growth of bacteria on coupons

### SEM visualization of biofilm formation on coupon slides

Scanning electron microscope observations confirmed biofilm formation on the surface of the coupons inoculated in storage containers with water from different systems. To provide the community with potential guidance in terms of maintenance of the water storage containers, preservation of the integrity of treated water quality and prevention of the development of biofilms on the surface of container-stored water, which led to the recontamination of treated water during storage, the treatment of water samples and the biofilm study were repeated twice after washing the storage containers and re-suspending the new coupons.

**Fig 6:**
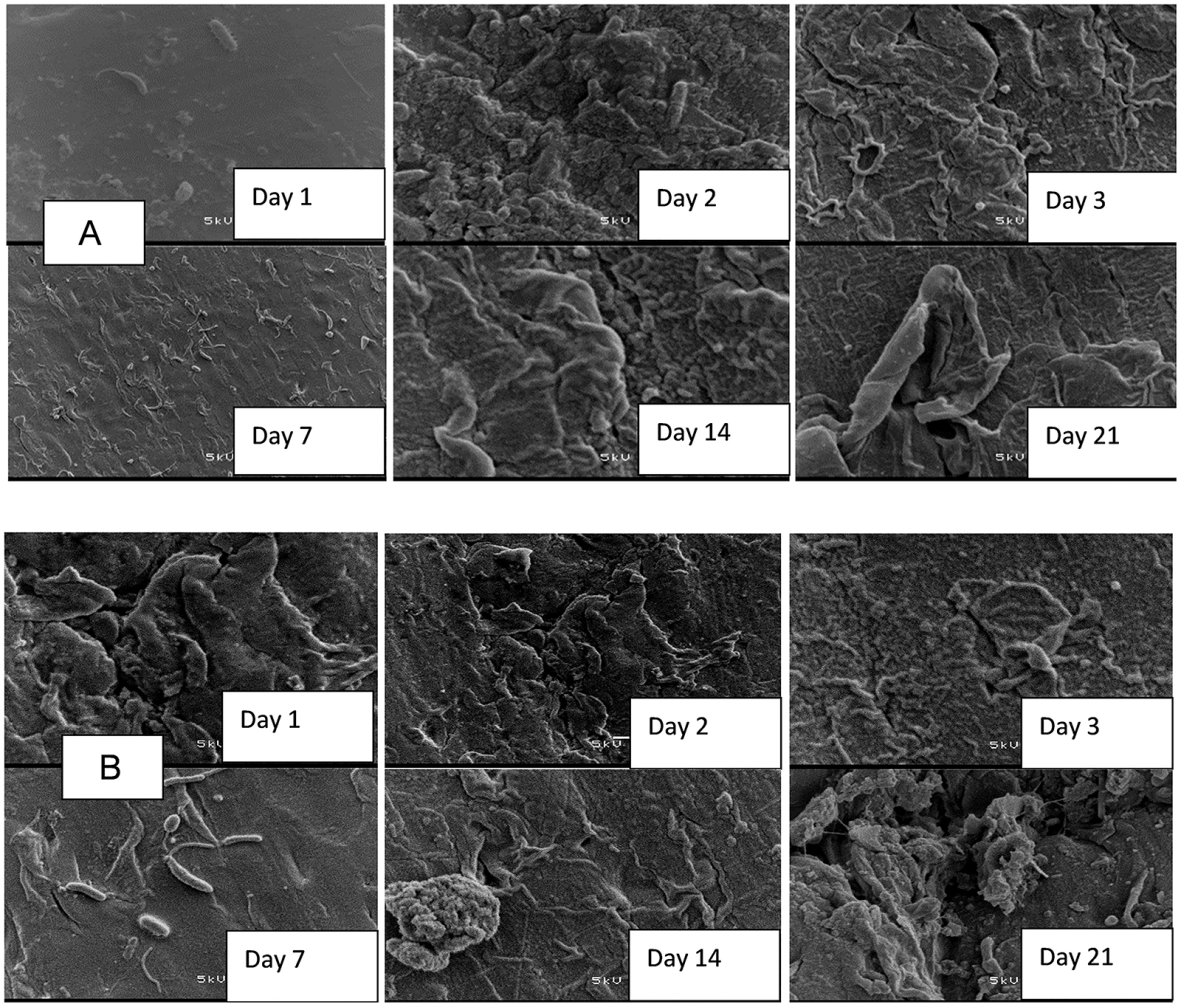
SEM images depicting attachment of microorganisms on the surface of coupons inoculated in untreated water (A: initial experiment, B: repeat).

**Fig 7:**
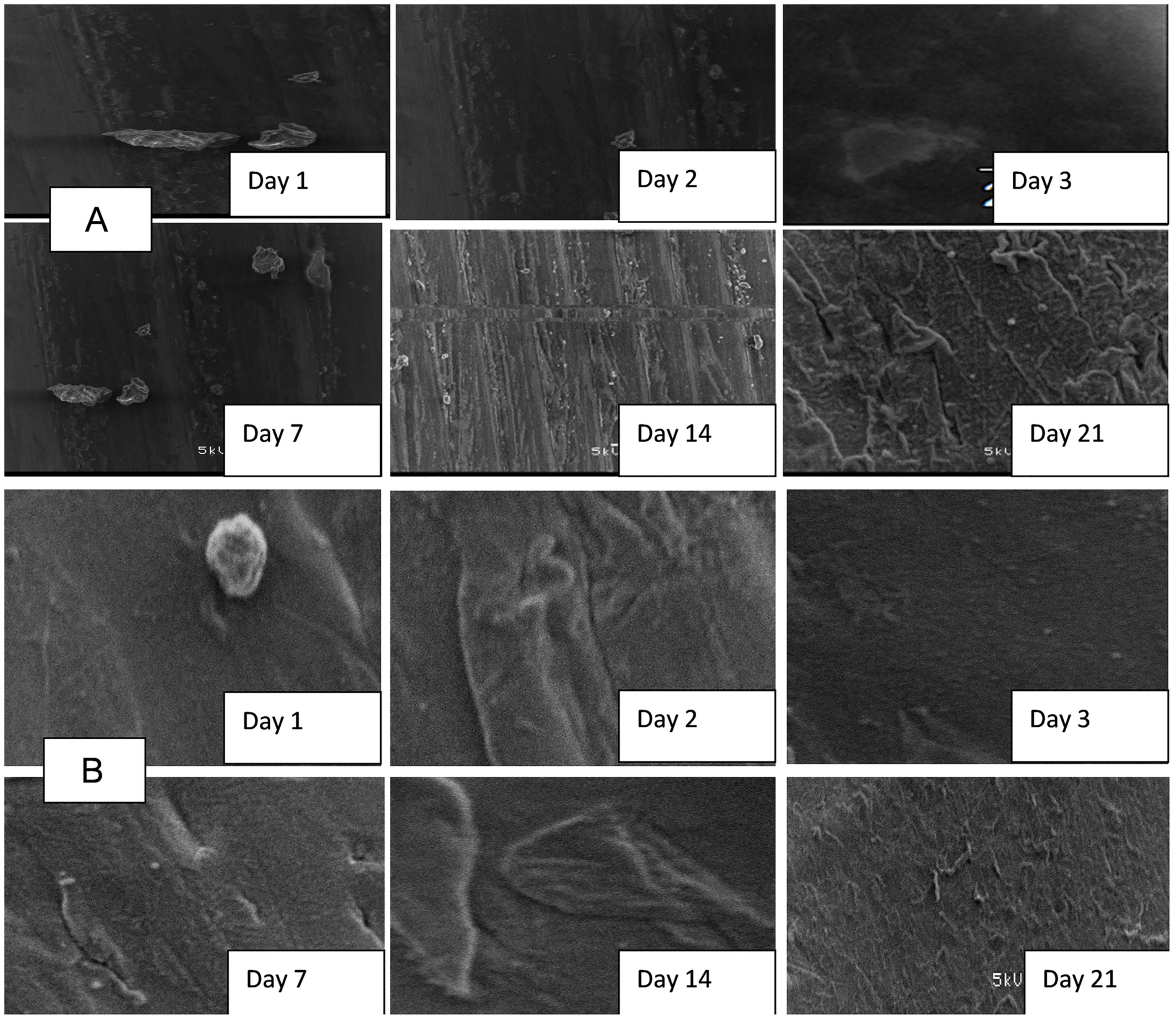
SEM images depicting attachment of microorganisms on the surface of coupons inoculated in treated water produced by SIPP-H system with an initial concentration of silver of 0.189 mg/L (A: initial experiment, B: repeat).

**Fig 8:**
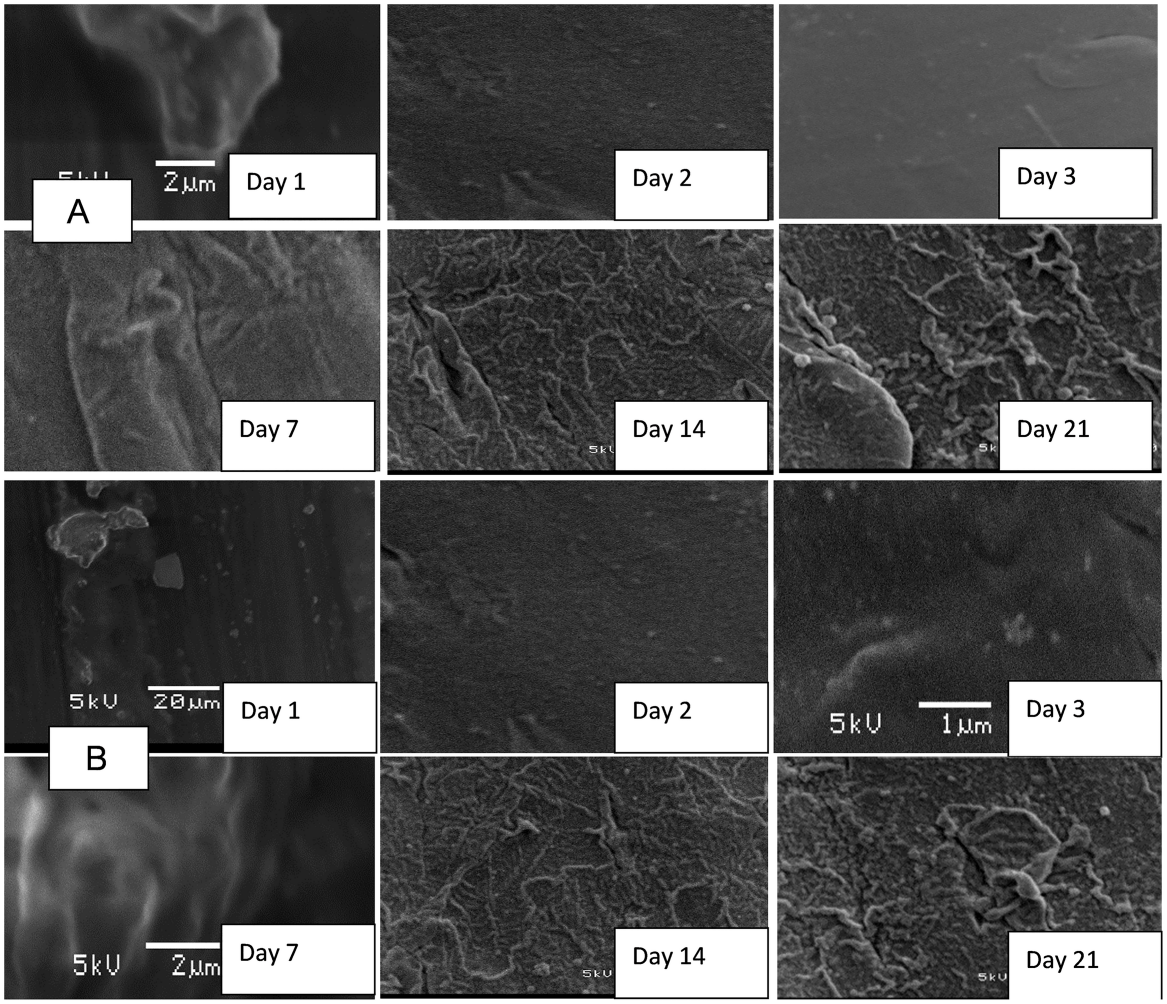
SEM images depicting attachment of microorganisms on the surface of coupons inoculated in treated water produced by SIPP-L system with an initial concentration of silver of 0.101 mg/L (A: initial experiment, B: repeat).

**Fig 9:**
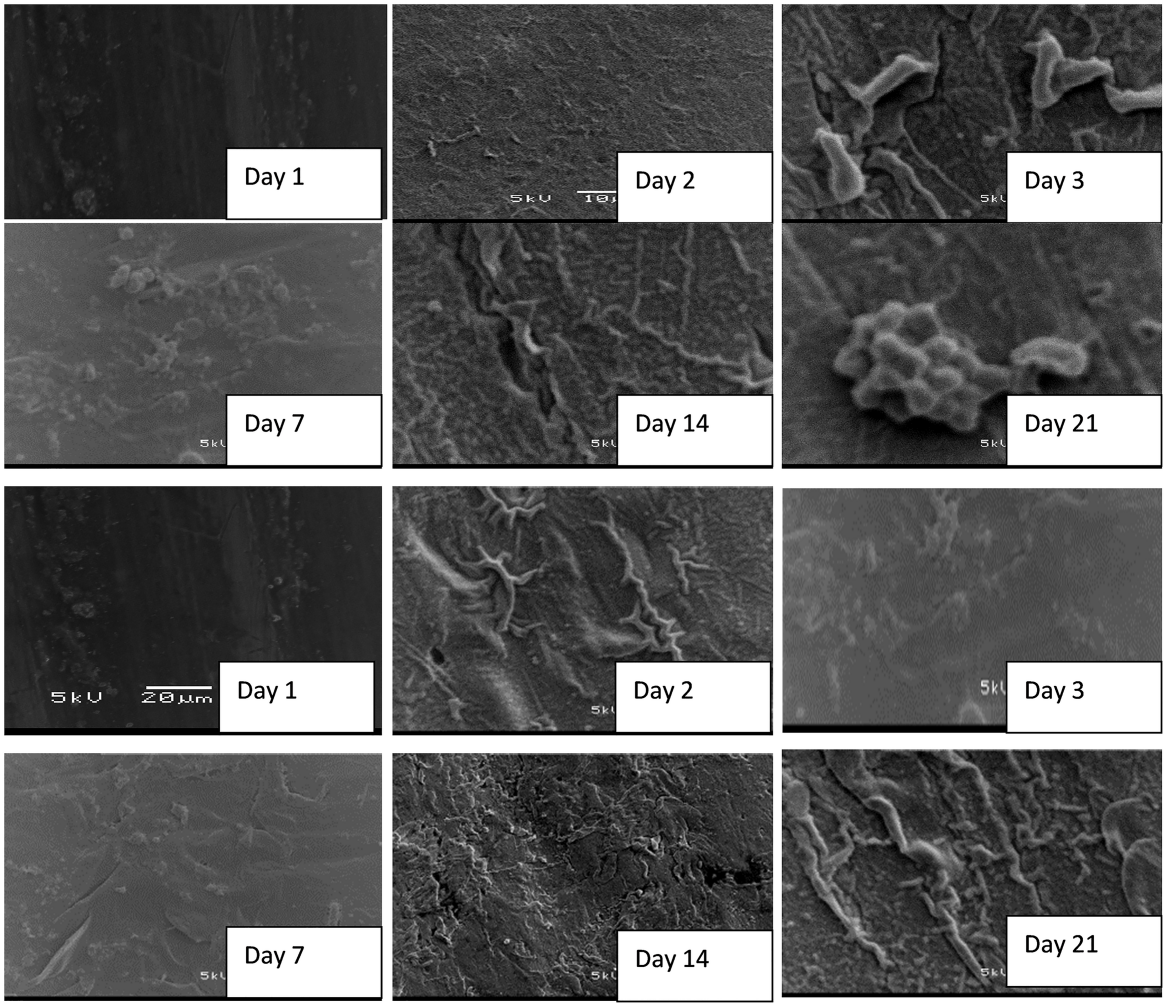
SEM images depicting attachment of microorganisms on the surface of the coupons inoculated in treated water produced by BSZ filter (filter without silver).

**Fig 10:**
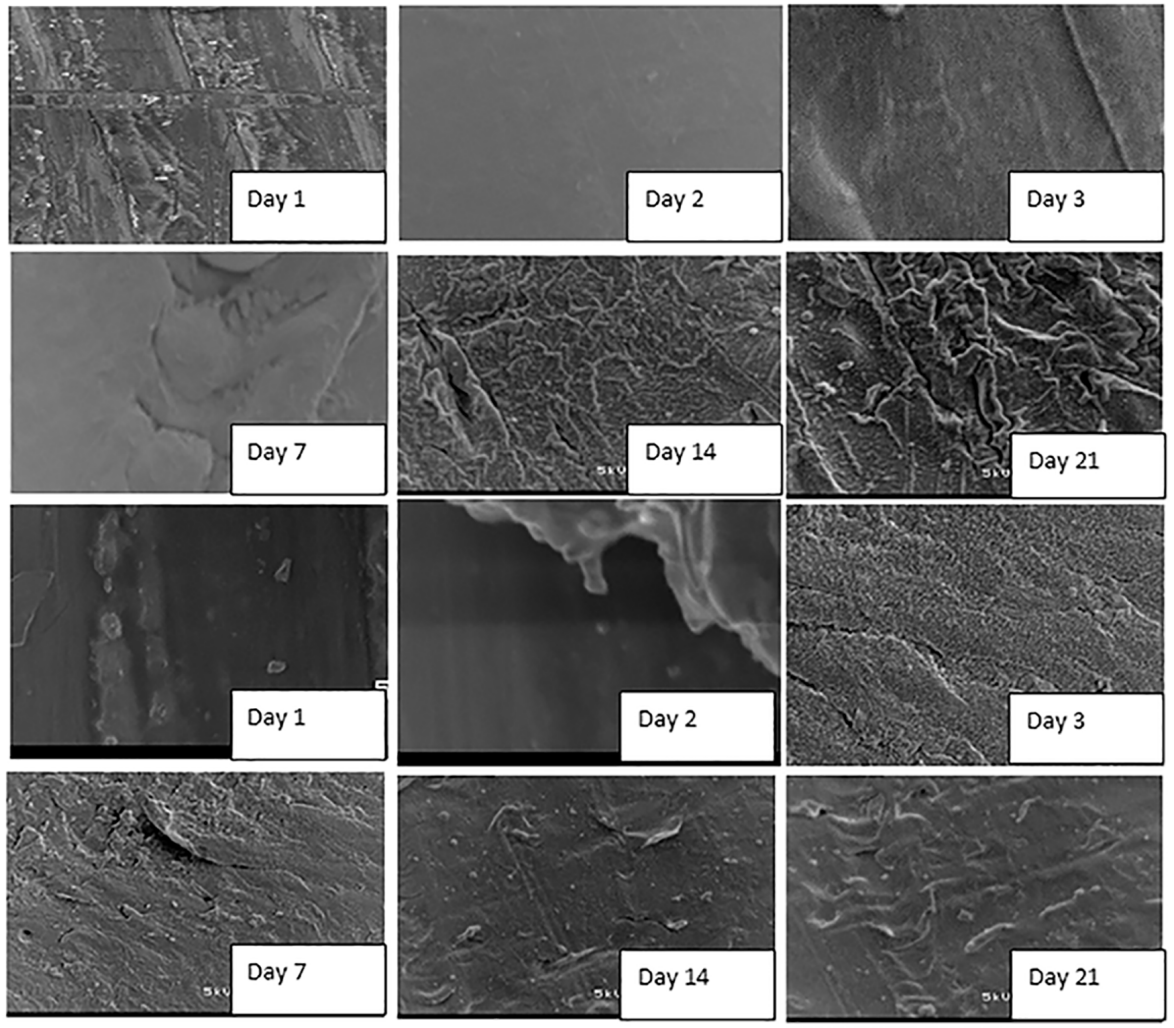
SEM images depicting attachment of microorganisms on the surface of coupons inoculated in treated water produced by BSZ-SICG system (after washing the storage container and re-suspension of new coupons, with a residual silver concentration of 0.045 mg/L).

Coupons inoculated in the storage containers with untreated water showed an initial development of biofilm within the first 24 h of storage, which drastically increased at the end of 21 days of the storage period Fig 6. Formation of biofilms on the surface of coupons inoculated in treated water produced by the SIPP-H filter (with an initial silver concentration of 0.189 mg/L) occurred after Day 21 Fig 7, while the evidence of biofilm formation on coupons inoculated in treated water produced by the SIPP-L filter (0.101 mg/L) was obvious from Day 14 and drastically increased by Day 21 Fig 8. Similar results were found during the initial and the repeat of the experimental study series with the SIPP filters as the leached silver concentrations remained constant in the treated water during the study period Fig 8. With the biosand filters, biofilm development was apparent from Day 2 on the surface of coupons inoculated in treated water produced by the BSZ filter (a biosand filter without silver) and progressively increased until Day 21 Fig 9. The same observations were also noted after washing the storage container and the re-suspension of the coupons. As to the BSZ-SICG filter, the formation of biofilms on the surface of coupons inoculated in treated water was apparent from Day 14 for the first experimental study series. However, after washing the storage container and re-suspending the new coupons, the biofilm development was observed earlier than expected, after Day 7 Fig 10.

## Discussion

Surface water is an important source of drinking water for populations around the world and it must be properly managed and protected[19]. It is imperative that drinking water that is meant for human consumption, complies with the SANS 241: 2015 standard set nationally [16] and internationally [20] in terms of physical, chemical and bacteriological parameters. These parameters have been used to determine the general quality of drinking water, worldwide. Results of the present study revealed that the surface water used by the Makwane community had mean alkalinity and temperature values within the limits set by [16] (pH 7.0–8.12), temperature; ≤ 25°C) for drinking water and domestic purposes. This range of alkalinity levels in drinking water reflects the presence of bicarbonate ion, which provides buffering action against acidic effects [21]. While the pH and the temperature of the Makwane water source cannot cause any public health risk, their surface water source was found to have high average turbidity levels, up to 260 NTU, which by far exceeded the limit (5 NTU) set by WHO guidelines. Turbidity is often caused by the presence of suspended clay, silt, organic matter, inorganic matter, plankton and other microscopic organisms [17]. The measurement of turbidity provides only an indication of the extent of the pollution of the water source [22]. Consequently, high turbidity levels in the water are associated with poor water quality and promote the survival of microorganisms[17]. This fact can clearly explain the high counts of indicator bacteria found in the Makwane Village surface water source Fig 4.

The heterotrophic plate count (HPC) test also called “total count” or “plate count” is used to estimate the total number of bacteria in a water sample that will develop into colonies during a period of incubation in a nutrient solution. This test detects a broad spectrum of bacteria including pathogenic, indicator and opportunistic pathogens[17]. The current study showed the number of HPC bacteria in Makwane surface water ranging between 4.4 and 4.3 Log_10_ CFU/100 mL, which by far exceeded the limit set by WHO 2015 for drinking water. According to UNESCO (2003) states that high HPC levels indicate the poor microbiological quality of drinking water [23]. Such elevated HPC levels may place the Makwane Village community, especially the children, elderly people and immuno-compromised individuals, at risk of contracting waterborne diseases when drinking water from this non-improved water source without any prior treatment. Furthermore, the Makwane Village main source of water supply did not only contain high numbers of heterotrophic bacteria, but also high counts of coliform bacteria group, which included total and faecal coliforms and *E*. *coli* Fig 4. This group of coliform bacteria also by far exceeded the limits set by the South African Water Quality Guidelines for human consumption (SANS 241: 2015), which is zero per 100 mL water sample for faecal coliform or for *E*. *coli* [16]. Such higher concentrations of coliform bacteria may be detrimental to the community as a whole, taking into account that surface water is the sole water source in this village. However, total coliforms comprise a heterogeneous group that includes bacteria from the genera *Escherichia*, *Citrobacter*, *Enterobacter*, *Klebsiella*, *Serratia* and *Rahnella*. Although most of these bacteria are members of the family Enterobacteriaceae, their presence may indicate the possible presence of pathogenic bacteria such as *Salmonella* spp. and *Shigella* spp., especially when detected in conjunction with other faecal coliforms [20]. The presence of faecal coliforms and *E*. *coli* indicates the contamination of the water bodies with human or animal wastes. Microorganisms present in this surface water source may cause acute infections characterized by symptoms such as diarrhoea, cramps, nausea, headaches and other symptoms. They may pose a health hazard to infants, young children, some of the elderly people and the immuno-compromised individuals [24].

Among pathogenic bacteria targeted during the study period, the presence of pathogenic *E*. *coli* strains (EHEC, EPEC, ETEC and EAEC) and *Salmonella Typhimurium* in the Makwane water source was confirmed using molecular techniques such as conventional PCR. It has been reported that pathogenic bacteria may occur in surface water in higher concentrations due to the practice of faecal excretion or by naturally occurring in the environment[24]. This can be applied to the surface water source of the Makwane Village as results of the survey revealed that open defecation is common in this community, with 40.9% of households lacking access to improved sanitation facilities and 96.5% disposing of wastewater in their yards. These practices clearly explain the prevalence of these pathogenic bacteria in their water source and corroborate the findings of other investigators who have pointed out that the presence of *Salmonella* spp. in surface water may be attributable to contamination from domestic sewage, agricultural pollution and storm water runoffs [25]. *Salmonella* spp. causes two types of salmonellosis: 1) typhoid fever and paratyphoid fever; and 2) gastroenteritis[24]. This implies that proper waste management education and personal hygiene may mitigate the incidence of gastroenteritis and typhoid/paratyphoid fever in the community. Although the *Vibrio* spp. and *Shigella* spp. were not confirmed by the molecular study, it is well known that their presence in water bodies indicates the contamination from birds, frogs, toads, and fish percent in aquatic environments [25]. *Vibrio* spp., especially *Vibrio cholerae*, has been implicated in the disease cholera in humans [26]. Thus, protecting the water sources would go a long way towards reducing the concentration of *Vibrio* spp. in the water bodies. The presence of *Shigella* spp. in surface water may be attributed to unsanitary conditions of the environment and secondary faecal contamination from intermediary sources. The implication of this is the risk of possible outbreak of shigellosis. Thus it is imperative to initiate a proper intervention programme aimed at educating the public about the risks of indulging in unsafe hygiene and sanitation practices. The detection of pathogenic bacteria in surface water in this area revealed an alarming situation in terms of contracting waterborne diseases. This study further highlights the need for implementation of household water treatment systems to mitigate the risk of exposure to bacterial pathogens.

Results of this study have shown that the improved SIPP and BSZ-SICG household drinking water treatment systems were able to deliver water that complied with the SANS 241 and WHO guideline limits in terms of physicochemical parameters analysed, especially turbidity Table 4. This finding is in agreement with the results of other studies that evaluated the efficiency of the initial version of these improved systems [5,6]. These authors also pointed out the significant positive correlation between the turbidity reduction in the treated water and the efficiency of these systems to produce drinking water of high microbial quality.

Although the present study focused only on the formation of biofilms on the surface of water storage containers, results revealed strong positive correlations between the water turbidity levels and the attachment of bacteria on the surface of the coupons inoculated in water storage containers Table 4 and Fig 5. The highest turbidity level in the untreated water Table 4 resulted in an early biofilm development on the surface of coupons within the first 24 h Fig 5. In contrast, a significant reduction of turbidity levels in treated waters after using the household drinking water treatment technology was found to be one of the factors which significantly contributed to the delay of biofilm development on the surface of coupons Table 4. Significantly lower turbidity levels were observed in water produced by SIPP filters compared to those in water produced by the biosand filter systems. The highest turbidity reductions, from an initial turbidity of 158–166 NTU to a final turbidity of 0.2–0.78 NTU, were obtained with the SIPP filters, a home water treatment technology that also exhibited biofilm development only after 14 days for the SIPP-L filter and after 21 days for the SIPP-H filter during treated water storage Figs 7 and 8 and Table 3. These results suggest that the SIPP filters, especially the SIPP-H filter, were able to inhibit biofilm formation on the surface of container-stored water up to Day 21 when the attachment of bacteria became apparent Fig 7. A similarity was found between the effectiveness of the SIPP-L filter and that of the BSZ-SICG filter in inhibiting the development of biofilms from Day 1 when compared to the effectiveness of BSZ filter system, a filter without silver Figs 7 to 9. Biofilm development occurred after 14 days on coupons inoculated in treated water produced by both SIPP-L and BSZ-SICG filters as these two systems did not show significant differences in the turbidity reduction during drinking water treatment. Very high turbidity levels in the water treated by BSZ systems resulted in early development of biofilms from 48 h of storage compared to the rest of the filters.

The silver nitrate (AgNO_3_) embedded in the clay of these treatment systems remained the most important factor that prevented early development of biofilms on coupons during the storage period. This implies a significant positive correlation between the concentration of the silver leached in the treated water and the inhibition of bacterial growth on coupons inoculated in water treated by SIPP and BSZ-SICG filters. A comparison between the untreated water stored in the control vessel and the SIPP/BSZ-SICG filters showed significant differences in biofilm development on coupons Figs 6 to 8. Significant differences in biofilm development were also shown between the BSZ filter without silver and the SIPP filter or BSZ-SICG filters. The leaching of the silver in treated water produced by these filters made these drinking water treatment technologies more effective in inhibiting biofilm formation on the surface of the container-stored waters. This also suggests that AgNO_3_ embedded in the filters ensured the removal of bacteria from water while inhibiting the attachment of these bacteria on the surface of the coupons which represented the storage containers. These findings confirm those of previous investigators who reported that the silver nanoparticles embedded in the clay during manufacturing contribute to high pathogen-removal efficiency[5,6,27]. Other studies have also revealed the effect of Ag in a water-purification application, irrespective of substrate and the antiviral and bacteriostatic properties of Ag ions [28]. A study by mwabi also demonstrated that only the Ag-impregnated pot was significantly more effective in removing *E*. *coli*, compared to the control pot without impregnated AgNO_3_ [5]. High performance of the SIPP and the BSZ-SICG filters in inhibiting the early development of biofilms on the container coupons was therefore attributed to the Ag nanoparticles embedded in the micropores of the clay pot of the former or the clay granular layer of the latter.

The bacterial removal efficiency of SIPP and BSZ-SICG home drinking water treatment technologies was also found to be dependent on the initial concentration of the silver embedded in the clay. Results of this study revealed significant differences between bacteria attached on the surface of coupons inoculated in treated water produced by the SIPP-H (with an initial silver concentration of 0.189 mg/L) and those attached on coupons inoculated in treated water produced by SIPP-L (0.101 mg/L AgNO_3_) or by BSZ-SICG (0.100 mg/L AgNO_3_). This implies that the higher the concentration of Ag embedded in micropores of the clay, the better the inhibition of biofilm formation on the container-stored water for a long period of the storage. However, the initial amount of silver that leached out into the treated water produced by the SIPP-H filter exceeded the recommended limit by [20] for drinking water, which is 0.1 mg/L. The fact is that to date, the only known health consequence of excessive silver intake is a condition known as argyria, which results in the discoloration of skin and hair by silver accumulation. According to [29], this condition may develop due to improper exposure to chemical forms of the element silver. This author further maintained that this condition has been linked to excessive consumption of medications containing silver and but not to the use of silver in drinking-water disinfection devices.

Strong positive correlations were found between the length of storage and bacteria attached on coupons, especially for those inoculated in untreated water Table 6 and Fig 5. This type of water source also resulted in a strong negative correlation for *Salmonella* spp. On the one hand, these findings imply a direct relationship between the length of storage and all target indicator bacteria (HPC, total coliforms, faecal coliform and *E*. *coli*) attached on coupons inoculated in untreated water Fig 5. On the other hand, there was an indirect relationship between the length of storage time of untreated water and attached *Salmonella* spp. This finding indicates that when the storage time was reduced, the attachment of *Salmonella* on the container-stored water increased and vice versa. There is a need for an effective barrier to prevent biofilm formation on the surface of household water storage containers. While the concentration of the silver leached out in treated water from SIPP and BSZ-SICG filters successfully delayed bacterial adhesion on the coupons, the long storage period factor could not be ignored as this factor reversed the efficiency of the systems. Strong positive correlations found between the length of storage time and limited indicator bacteria (HPC and total coliforms) attached on coupons inoculated in treated water imply that only HPC and total coliform bacteria had the capability to grow on the surface of plastic containers during an excessively long period of drinking-water storage. The storage period varied between 14 and 21 days for SIPP-L and between 7 and 21 days for the BSZ-SICG filters Fig 7. This significant difference in the growth of bacteria on the surface of container-stored water was due to a progressive decrease in silver concentration that occurred in the water treated by the BSZ-SICG filter during the storage period, whereas the silver concentration was found to remain constant in water treated by the SIPP filter. The long storage period automatically led to the recontamination and deterioration of the treated water quality. Findings of this study are in agreement with those of [14], who also found that the length of storage time resulted in re-growth and survival of indicator bacteria on the surface of household containers used for the storage of drinking water in rural communities of South Africa. As demonstrated by these authors, both polyethylene and galvanized steel containers supported the persistence of indicator bacteria on the surface of the container-stored water due to the deterioration of the quality of the treated water during storage. To prevent the deterioration of treated water quality in storage containers, this study suggests that all containers should be washed after 7 days when using BSZ-SICG filters and after 14 days when using the SIPP filters during the storage period.

## Conclusions and recommendations

Surface water, which is the sole water source for the rural community of the Makwane Village, is characterised by pH and temperature values, which are within the limits set by the South African National Standard [16] and the World Health Organization. In contrast, the turbidity values of this water source far exceeded the recommended limits. Moreover, the water source is heavily contaminated and harbours unacceptably high counts of indicator bacteria such as HPC, faecal coliforms and *E*. *coli*. Prior to treatment, molecular studies revealed the presence of all pathogenic strains of *E*. *coli* (EHEC, EPEC, ETEC and EAEC) and *Salmonella* Typhimurium in this surface water source used by the community for multiple purposes, including drinking and cooking. The absence of these target organisms after the treatment of surface water indicates the effectiveness of the BSZ-SICG and SIPP filters in producing safe drinking water and providing an effective barrier for the inhibition of biofilm formation on the surface of household water storage containers. Factors such as the turbidity of the water, the initial silver concentration embedded in micropores of the clay during manufacturing and the length of storage time play a major role in preventing the adhesion of bacteria on the inner surface of the storage containers. To effectively prevent the re-growth of bacteria on the surface of the storage containers and the deterioration of the treated water quality, this study recommends that the storage containers be washed with disinfectant within 14 days of the storage of the water treated with SIPP filters and within 7 days for the water treated with BSZ-SICG filters.

## Supporting information

S1 File Consent form. (DOCX)
