# Supplementary material for: Inhibition of biofilm formation on the surface of water storage containers using biosand zeolite silver-impregnated clay granular and silver impregnated porous pot filtration systems
Source: PLoS One. 2018 Apr 5;13(4):e0194715. doi: 10.1371/journal.pone.0194715 (PMC5886460; doi:10.1371/journal.pone.0194715)
Supplement: S1 File — (DOCX) [file pone.0194715.s001.docx]

**Appendix A**

**Informed consent form**

(Form for research subject’s permission, must be signed by each research subject, and must be kept on record by the researcher)

Title of the research project: **Implementation of cost-effective decentralized household water treatment systems for the production of adequate clean and safe drinking water in rural communities**

I ……………………………. hereby voluntarily grant my permission for participating in the project as explained to me by…………………………….

The nature, objective, possible safety and health implications have been explained to me and I understand them.

I understand my right to choose whether to participate in the project and that the information furnished will be handled confidentially. I am aware that the results of the investigation may be used for the purpose of publication.

Upon signature of this form, you will be provided with a copy.

Signature……………………………………………………… Date……………………………………….

Witness…………………………………………………………… Date……………………………………

Researcher……………………………………………………… Date…………………………………………
